# Case Report: Differential lung ventilation with jet ventilation *via* a bronchial blocker for a patient with a large thoracogastric airway fistula after esophagectomy

**DOI:** 10.3389/fsurg.2022.959527

**Published:** 2022-11-08

**Authors:** Wenqi Wu, Sibei Li, Xiuling Song, Xia Wang, Yong Wang, Chengyi Cai, Jiyong Wang, Yuhui Li, Wuhua Ma

**Affiliations:** ^1^ Department of Anesthesiology, The First Affiliated Hospital of Guangzhou University of Chinese Medicine, Guangzhou, China; ^2^ Department of Thoracic Surgery, The First Affiliated Hospital of Guangzhou University of Chinese Medicine, Guangzhou, China

**Keywords:** thoracogastric airway fistula, airway management, bronchial blocker, single lumen endotracheal tube, case report

## Abstract

**Background:**

A thoracogastric airway fistula (TGAF) is a rare and potentially fatal complication of esophagectomy for esophageal and cardia carcinomas. Isolation of the fistula and pulmonary separation is necessary during the surgical repair of a tracheal fistula. However, currently, the reported airway management techniques are not suitable for patients with a large TGAF. This case study presents an alternative technique for performing differential lung ventilation in a patient with a thoracogastric airway fistula.

**Case presentation:**

A 70-year-old man was diagnosed with a thoracogastric airway fistula situated above the carina after esophagectomy, and a thoracoscope-assisted repair of the fistula and pectoralis major myocutaneous flap transplantation were scheduled. The patient could not tolerate one-lung ventilation and the complex intubating operation due to aspiration pneumonia and the size (3.5 cm × 1.7 cm) of the fistula. We, therefore, performed differential lung ventilation in which an extended 6.5#single-lumen endotracheal tube was inserted into the left main bronchus and a 9Fr bronchial blocker was placed in the right main bronchus by using the video-flexible intubation scope. The right lung was selectively inflated with jet ventilation, while positive pressure ventilation was maintained through the left endotracheal tube. The value of SPO2 remained above 95% throughout the operation.

**Conclusion:**

For patients with a large thoracogastric airway fistula, differential lung ventilation of a combination of positive pressure ventilation and jet ventilation is useful. Inserting an extended single-lumen endotracheal tube into the left main bronchus and a bronchial blocker into the right main bronchus could be another way of providing differential ventilation for patients with a large thoracogastric airway fistula.

## Introduction

A thoracogastric airway fistula (TGAF) is a rare but seriously life-threatening complication of esophagectomy for esophageal and cardia carcinomas, and effective airway management is challenging due to the poor clinical status of patients and complex anomalies (1). Treatment can be either conservative or includes endoscopic and surgical repair (2). Pulmonary separation and prevention of aspiration is required in the surgical repair of the fistula in the lower trachea. A small number of case reports have detailed the use of one-lung ventilation or differential lung ventilation, achieved by inserting a double-lumen endotracheal tube (DLT) or two single-lumen endotracheal tubes (SLTs), in tracheoesophageal fistula (TEF) repair, but there are limited data on a large TGAF (3, 4). In a patient with a large TGAF above the carina, a DLT may encroach on the tracheal defect, interfering with the reparation. Bilateral single-lumen endotracheal tubes may cause mucosal damage and migrate out of position. Here, we report the differential lung ventilation using a combination of positive pressure ventilation in the left lung and jet ventilation *via* a bronchial blocker (BB) in the right lung for a patient with a large TGAF.

## Case description

**Figure 1:**
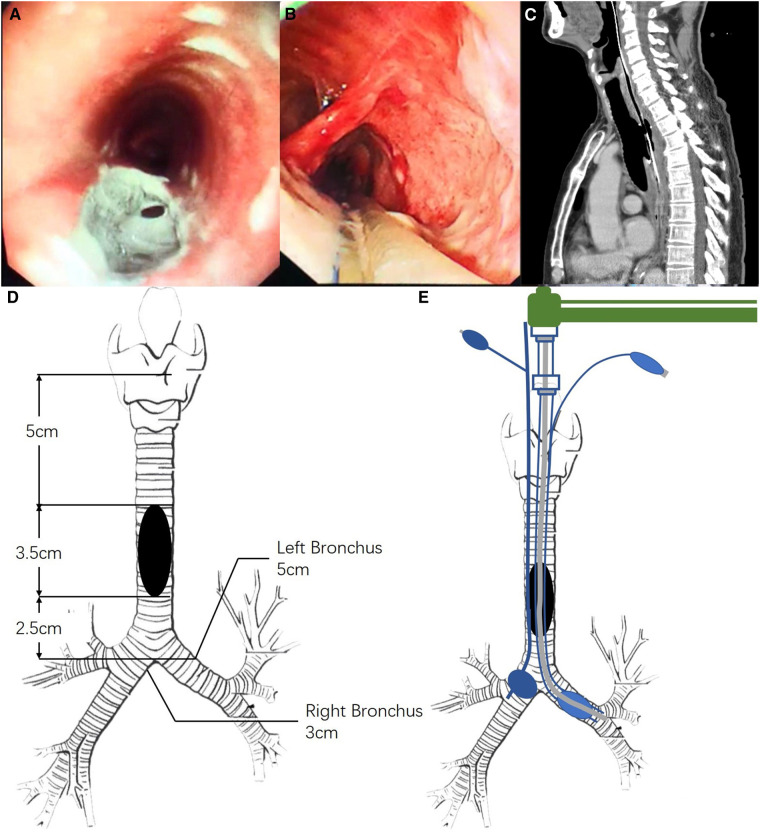
Characteristics of the thoracogastric airway fistula. (**A**) Gastroscope view of the thoracogastric airway fistula; (**B**) bronchoscope view of the thoracogastric airway fistula; (**C**) computed tomography of the thoracogastric airway fistula; (**D**) length of the tracheal fistula; and (**E**) left main bronchus intubation *via* an extended Parker flex-tip tube, and the right main bronchus with a balloon-tip bronchial blocker.

A 70-year-old man (60 kg, 170 cm) was diagnosed with a large thoracogastric airway fistula after esophagectomy. Two months earlier, he underwent esophagus tumor resection and cervical esophagogastric anastomosis *via* the three incisions of thoracoscopy through the right chest, abdomen, and left neck for moderately differentiated squamous cell carcinoma. After surgery, he developed repeated expectoration and coughing after eating. The use of a bronchoscope and gastroscope confirmed a large fistula (3.5 cm × 1.7 cm) 5 cm below the glottis, and its inferior margin was 2.5 cm above the carina (Figure 1). Preoperative PaO_2_ was 101.8 mmHg breathing room air. A chest radiograph revealed a right pneumothorax, bilateral lung infection, and basal pleural effusion. After administration of antibiotics, gastrostomy feeds, and total parenteral nutrition for a month, he was planned for a repair of the fistula and pectoralis major myocutaneous flap transposition under the right posterolateral thoracotomy approach and a thoracoscope-assisted one.

Computed tomography showed the fistula with upper and lower edges at 5 cm and 2.5 cm away from the glottis and the carina (Figure 1D). The left and right main bronchus length was 5 and 3 cm. After consultation with surgeons, we decided to intubate a 6.5 extended Parker Flex-Tip Tube (total length 34 cm, Wellead®; Well Lead Medical Co., Ltd., Guangzhou, China) into the left main bronchus and a 9Fr bronchial blocker (Wellead®; Well Lead Medical Co., Ltd.) to occlude the right main bronchus (Figure 1E). Intraoperative monitoring included electrocardiography, capnography, blood oxygen saturation, and invasive blood pressure. Cardiopulmonary bypass was on standby in case oxygenation could not be maintained during the operation.

The patient spontaneously breathed in the operation room, was given supplemental oxygen, and inhaled 2% lidocaine *via* a high-flow nasal cannula (HFNC, 20–35 L min^−1^, Fisher&Paykel Healthcare). Under suitable sedation using dexmedetomidine (4 μg/kg/h for 15 min) and sufentanil (5 μg), topical oral cavity and posterior pharynx anesthesia were achieved with a 2.4% lidocaine spray, glottis anesthesia with 2% lidocaine *via* an epidural catheter through the working channel of a video-flexible intubation scope (VFIS), and subglottic with 2% lidocaine (2 ml) *via* a cricothyroid membrane puncture injection. Following satisfactory topical anesthesia, a cuffed 6.5 extended Parker Flex-Tip Tube was guided into the left main bronchus under the VFIS with a background infusion of 50 mg propofol. After isolating left lung ventilation and correcting the cuff position with VFIS examination and auscultation, general anesthesia was induced and maintained with propofol, sevoflurane, remifentanil, and vecuronium. An additional 10 cm extension limb (7.5# single-lumen tube, Wellead®; Well Lead Medical Co., Ltd., cut short into 10 cm) was used for connecting the circuit and the endotracheal tube (Supplementary Figure S1). Then, 9Fr BB was passed through the glottis under a video laryngoscope. The balloon was placed toward the anterior tracheal wall and turned right after passing through the glottis. The BB position was evaluated by VFIS observation and auscultation under jet ventilation (pressure: 10–15 PSI, frequency: 12 times per minute, at the head-low position, and the balloon deflated). One-lung ventilation was initiated at a fraction of inspired oxygen (FiO2) of 1.0 with volume control ventilation (tidal volume 360–420 ml, respiratory rate 12/min, peak inspiratory pressure 30 cm H_2_O, I:E 1:1.5). After establishing the airway, a central venous catheter was placed through the right internal jugular vein (Table 1).

**Table 1:** Induction timeline.

Time	Event
11:40	Entering the OR
12:05	HFNO (20–35 L min^−1^, inhaled O2, and 2% lidocaine)
12:10	Administration of dexmedetomidine and sufentanil
12:25	Topical anesthesia for glottis under a VFIS
12:40	Cricothyroid membrane puncture injection (2% lidocaine)
12:44	Injection of propofol (50 mg)
12:45	Insertion of a 6.5 extended Parker flex-tip tube into the LMB under a VFIS
12:50	Start of induction (propofol, sevoflurane, remifentanil, and vecuronium)
13:00	Insertion of a 9Fr BB into the RMB under a video laryngoscope
13:08	Puncture and catheterization of the left radial artery
13:20	Puncture and catheterization of the right internal jugular vein
14:23	Start of the surgery

OR, operation room; HFNO, high-flow nasal cannula oxygen therapy; VFIS, video-flexible intubation scope; LMB, left main bronchus; BB, bronchial blocker; RMB, right main bronchus.

The operation was performed in the left lateral position. During surgery, jet ventilation under the left lateral decubitus position with head down after airway clearance was performed to verify air leakage in the right lung. PaO_2_ was between 139 and 291 mmHg throughout the operation, while ETCO2 was between 36 and 53 mmHg. The value of SPO2 remained above 95% throughout the operation. The operation lasted for 6 h. The right lung was expanded *via* jet ventilation when the repairs were completed. After full-airway clearance and BB removal, the endotracheal tube was withdrawn to the trachea. The cuff was placed above the anastomosis and below the glottis under the guidance of a VFIS. Blood gas analysis showed a PaO2 of 282 mmHg. The patient was transferred to the intensive care unit uneventfully. A 12-h postoperative bedside chest radiograph showed no significant gastrointestinal inflation (Supplementary Figure S2).

## Discussion

A thoracogastric airway fistula after esophagectomy is a rare disease with a reported incidence of 0.8%–1.5%. The main treatment options include surgical repair, stent implantation, and conservative treatment (2). Conservative treatment was rejected as the fistula size (3 cm) was considered too large to heal naturally. Regarding the selection of surgical methods, some reports on new devices and methods of stent placement have shown their advantages and potential for treating tracheoesophageal fistulas, including degradable stents, Amplatzer® devices, and endobronchial one-way umbrella-shaped valves. However, degradable stents are suitable for patients who do not need to permanently dilate the stenosis or cover the fistula (e.g., infants and children) (5). The Amplatzer® device is an alternative for patients who cannot receive surgical repair and place clips due to fibrosis around the fistula (6, 7). In addition, the model of the Amplatzer® device was limited by fistula size in this patient, and a huge disc may reduce the airway cross-sectional area and prevent the closure of the fistula (8). The endobronchial one-way umbrella-shaped valve is mostly used in patients with bronchial pleural fistula (BPF), which is suitable for small fistulas (9). No relevant use in TEF patients has been reported. A systematic review analyzed the outcomes of surgical vs. non-surgical procedures in patients with tracheogastric fistula. Non-surgical treatment can improve the healing rate of the initial fistula, while surgically treated patients have better survival rates. Considering that the patient's fistula occurred for a short time and was eligible for one-stage repair without stenosis and obstruction, the thoracic surgery department finally decided to perform a one-stage repair of the fistula in this patient.

There are few reports about anesthesia and airway management of the surgical repair of a thoracogastric airway fistula. The goal of anesthetic management is to ensure good ventilation, seal the fistula to avoid aspiration and gastric inflation, and minimize the risk of airway injury. For the fistula in the lower trachea, a pulmonary isolation technique is required, and several different pulmonary separation techniques have been reported, including the insertion of a DLT (3), intubation of an SLT into the bronchus (10), and insertion of two SLTs into the bilateral bronchus (4). However, none of them can meet the above requirements simultaneously (Table 2).

**Table 2:** Qualitative comparison of different lung separation techniques for the fistula in the lower trachea and carina.

Airway management	Ventilation management	Ventilation effect	Airway sealing	Surgical exposure	Risk of tracheal injury	Difficulty
DLT in LMB	OLV	+	+	++	+	+
SLT in LMB	OLV	+	+	++	Rare	
BBI	TLV	++	++	+	++	++
SLT in LMB and BB in RMB (Our method)	OLV and JV	++	++	++		+

DLT, double-lumen endotracheal tube; LMB, left main bronchus; SLT, single-lumen endotracheal tube; BBI, bilateral bronchial intubation; BB, bronchial blocker; OLV, one-lung ventilation; TLV, total-lung ventilation; JV, jet ventilation.

Before the operation, the anesthesiologist and the surgery team discussed the anesthesia strategy. One option was to insert a DLT into the left main bronchus. This option was vetoed because the size of the bronchial cuff of the DLT is only 2 cm [Shiley (Covidien, Mansfield, MA, United States) DLT, left 37 Fr/Ch, endotracheal diameter 12.3 mm], which failed to close a 3.5 cm fistula. Furthermore, a DLT may encounter difficulties in intubation and its outer diameter may rupture the fistula during intubation. Some colleagues suggested another method that inserts an SLT into the left main bronchus, an alternative technique for children without a suitable size of the DLT (11). Compared with a DLT in the same inner diameter, the outer diameter of an SLT is smaller and easier to operate. Still, this option was also vetoed as it would not resolve the issue of aspiration in the right lung, and the right lung cannot be ventilated.

A third approach was intubation with two SLTs for both lungs as discussed in the article (4, 12–14). However, we thought that it is challenging to insert the second SLT into the right bronchus in such a large fistula and the patient can be at risk of tracheal injury. Total-lung ventilation may significantly decrease surgical exposure. One more option is extracorporeal membrane oxygenation (ECMO), which can ensure stable gas exchange and which avoids the risk of complex airway operations. Nevertheless, it is inevitable to aspirate blood and fragments without occlusion of the airway, and systemic anticoagulation will lead to vascular injury, infection, bleeding, and other complications. A recent meta-analysis confirmed that ECMO with low-dose heparin could reduce surgical site bleeding without increasing postoperative mortality and bleeding. The current reports of ECMO for tracheoesophageal fistula are mostly seen in pediatric and infant patients, as an alternative to the inability to insert a double-lumen tube. Several reports of ECMO for TEF anesthesia management in adult patients show its potential as a final solution for various types of airway intubation failure in these cases. Because of the high cost, this option was rejected by the patient. Therefore, we finally decided to insert a small (6.5#) SLT into the left main bronchus for positive pressure ventilation and inserted a 9Fr BB into the right main bronchus and connected the jet ventilation device. Jet ventilation is performed through BB when hypoxemia occurs during the operation. Some authors believe that the length of the Mallinckrodt microlaryngeal tube (MLT) and the design of the small cuff are suitable for endobronchial intubation. Nevertheless, we considered that the fusiform-shaped cuff of MLT might cause tube migration after inflation. Finally, left one-lung ventilation was accomplished *via* an extended single-lumen 6.5# SLT with a 10 cm extension limb (7.5# SLT cut short into 10 cm). No displacement was detected after intubation.

We used high-flow nasal oxygen (HFNO) as an enhanced version of the nasal oxygen catheter for preoxygenation instead of using a mask. A nasogastric tube and a nasojejunal nutrition tube were placed through the patient's right nostril. The mask does not seal well and most oxygen will escape from the mask than get inhaled. Mask-positive ventilation will impede the use of a VFIS and increase the risk of reflux and aspiration in patients with such a fistula. Alternatively, using HFNO ensures that the patient will receive adequate oxygen for an extended period to complete full-airway topical anesthesia. Usually, the rate should be more effective, up to 70 L/min. However, we observed a significant increase in coughs of the patient at 70 L/min rates, so we gradually lowered the flow rate of HFNO. Considering that the patient’s level of comfort and high flow may increase the risk of reflux aspiration, the rate of 30–40 L/min was proved effective.

Jet ventilation was prepared for total-lung ventilation for emergency conversion. Considering that the out-sync ventilation of two lungs might cause changes in hemodynamics, jet ventilation (pressure: 10–15 PSI, frequency: 12 times per minute) through the bronchial blocker was prepared as an alternative measurement to achieve total-lung ventilation. The jet ventilation was made through the port of the blocker. Before using the jet ventilation, the airway secretion was properly cleared, the position was changed to head-low, and the blocker balloon was loosened to avoid reflux and jet ventilation–associated barotrauma. Other scenarios using jet ventilation included the right lung ventilation test after bronchial blocking, the test for air leakage of the right lung, and the postoperative right lung expansion test.

## Conclusions

We described differential lung ventilation by combining positive pressure ventilation and jet ventilation to manage a patient with a large thoracogastric airway fistula above the carina. Inserting an extended single-lumen endotracheal tube into the left main bronchus and a bronchial blocker in the right main bronchus could be another way of providing differential ventilation for these patients. The advantages of this case study are as follows: (1) preoperative measurement of trachea and lesion prevented repeated intubation and sequent secondary injury; (2) a high flow provided continuous oxygenation during topical anesthesia and awake intubation; (3) a thorough topical anesthesia of the airway *via* multiple techniques increased the success rate of intubation; (4) left lung separation and ventilation were achieved *via* bronchial intubation using a single-lumen endotracheal tube; (5) right lung separation was ensured by using a balloon-tip bronchial blocker; (6) right lung ventilation and postoperative inflation were implemented through jet ventilation by using a bronchial blocker.

## Data Availability

The original contributions presented in the study are included in the article/Supplementary Material, further inquiries can be directed to the corresponding authors.
