# Supplementary material for: Case Report: Differential lung ventilation with jet ventilation via a bronchial blocker for a patient with a large thoracogastric airway fistula after esophagectomy
Source: Front Surg. 2022 Nov 8;9:959527. doi: 10.3389/fsurg.2022.959527 (PMC9679631; doi:10.3389/fsurg.2022.959527)
Supplement: Supplementary file 1 [file Datasheet1.docx]

Supplementary Material
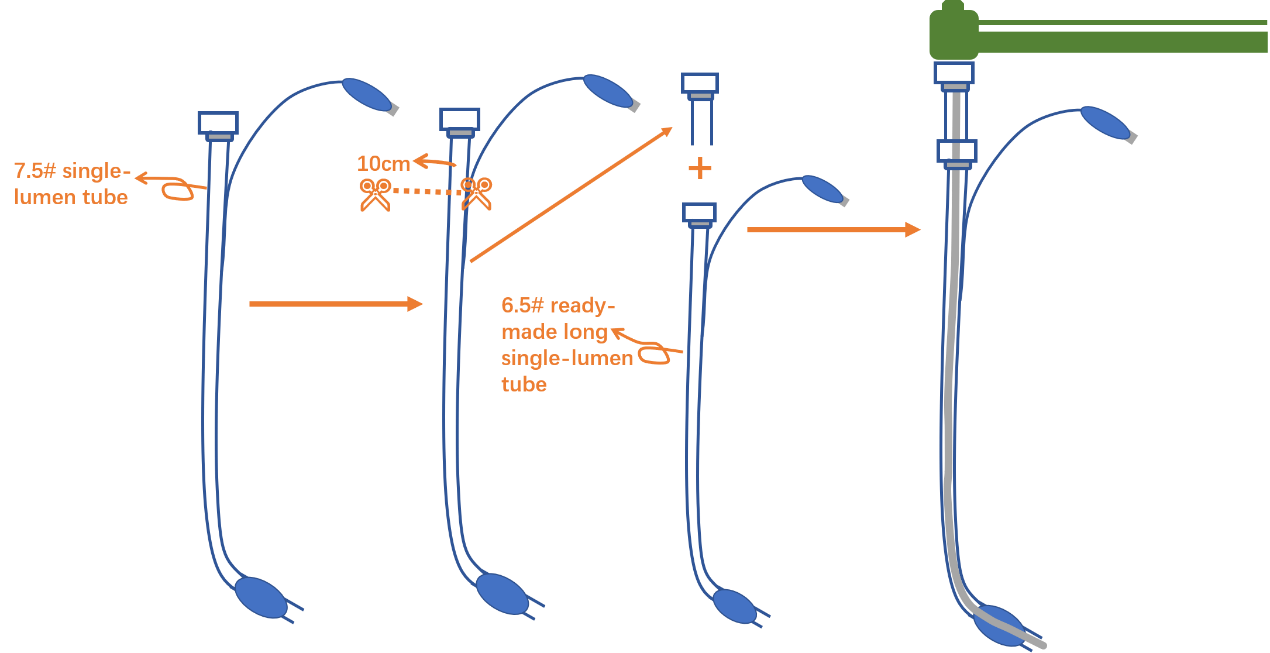


**Supplementary Figure 1.** Schematic diagram of connecting two ETTs

**
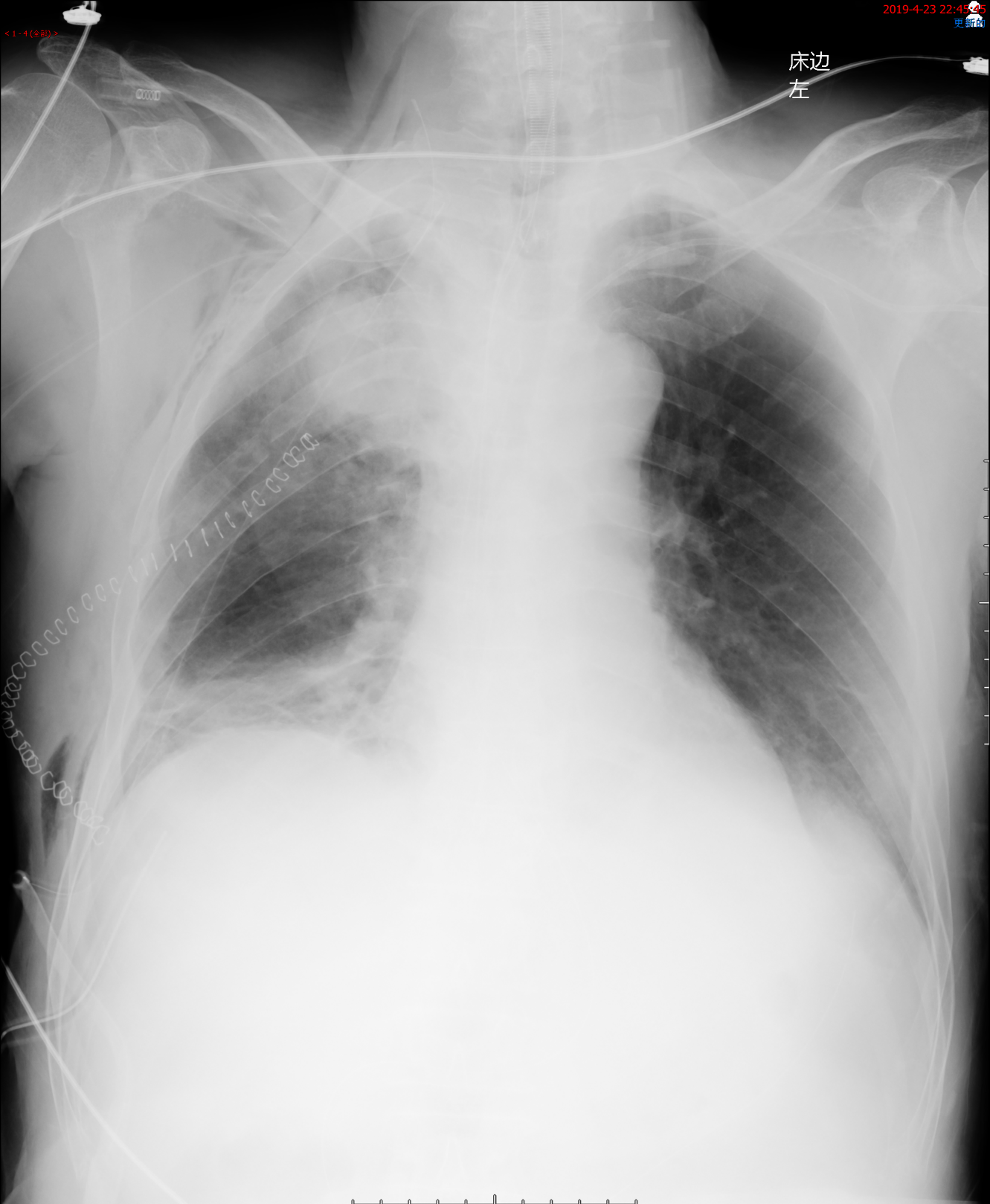
**

**Supplementary Figure 2.** 12-hour postoperative bedside chest radiograph
